# Notes and News

**Published:** 1895-05-25

**Authors:** 


### Notes and News.

(Collected and Communicated.)

### HOSPITAL MEETINGS.

Royal Eye Hospital.—Festival Banquet, Tuesday, June 18th, at Hôtel Métropole.

Middlesex Hospital.—Quarterly Court, Thursday, May 30th.

National Society for the Employment of Epileptics.—Annual Meeting at 12, Buckingham Street, Strand, on Monday, May 27th, at five o'clock.

West London Hospital.—Meeting in aid of the Charity at Devonshire House on Wednesday, June 12th, at four p.m.

In Switzerland, the Canton of Berne has just abolished compulsory vaccination.

A disease allied to foot and mouth disease has made an invasion into the City of Berlin.

The Viennese medical profession propose commemorating the jubilee of the Austrian Emperor by the erection of a very large hospital for children.

The career of doctors appears to grow more difficult as time goes on. In North Carolina medical men have to pay an annual tax of £2 for the privilege of following their profession.

The medical profession is becoming very popular amongst the ladies in America. One quarter of the students at the Johns Hopkins Medical School are women.

A new morgue is to be erected in Paris, in addition to the present building. It is proposed that the bodies shall be kept for nearly three months for purposes of identification.

A bazaar in aid of the Royal Westminster Ophthalmic Hospital was opened last week by Princess Henry of Battenberg. The work of the hospital has outgrown the limited space set aside for it, and new buildings are contemplated.

American laws are known to render divorce in many respects an easy matter, but a recent attempt to remove yet another barrier, by permitting divorce to be procurable after one or other of two married persons has been proved insane during five years, was promptly resented, and the Bill did not pass.

It is announced by the committee of the Paddington Green Children's Hospital that H.R.H. the Duchess of Teck has consented to receive purses of £5 and upwards on the occasion of the opening of the new building on Monday, July 1st; £3,000 is still required for the rebuilding and furnishing fund. The secretary will be glad to receive the names of intending collectors.

The Royal visit to Sheffield was a great success. The weather was brilliant, and the whole town *en fête*. The Duke of York, in declaring the new wing of the hospital open, announced that the Queen had approved of the institution being known as the Royal Sheffield Hospital for the future. His Royal Highness also announced the result of the collections in honour of the occasion, which amounted to the splendid sum of £29,873. The present accommodation of the hospital will now be increased from 100 to 160 beds.

At the recent annual meeting of the Devon and Exeter Hospital a resolution was carried "that none of the capital funds of the hospital be spent by the committee without the consent of the governors, and that all legacies and benefactions of £25 and upwards be treated as capital." Hitherto some portion of the legacies received every year have been spent, but it was argued that whilst capital was thus drawn upon the public could not be convinced of the real needs of the hospital. Many important alterations are needed within the hospital, which are estimated to cost £12,000. Under £4,000 is at present forthcoming.

Mr. B. Burford Rawlings is an ideal hospital secretary in many ways. He is always at work for his institution, and fortunately for it, he is a writer who possesses originality and power. His latest work is a powerful appeal with illustrations of the National Hospital for the Paralysed and Epileptic, entitled "Things Seen are Greater than Things Heard," which has already produced over one thousand pounds for the funds of the institution he so ably directs, and we trust it may produce as much again. As the late Sir William Gull once said, it is of the greatest importance to the public to know that the Queen has given her confidence to the National Hospital for the Paralysed and Epileptic, because the public may in consequence give their money to it without stint or hesitation.

We have received the fifty-second annual report of the Mutual Life Insurance Company, of New York, 17 and 18, Cornhill, London, E.C., which shows substantial progress, there having been an increase to the extent of upwards of £103,000,000 in insurance and annuities in force, as compared with ten years ago. The Mutual Life Insurance is deservedly famous for the variety and ingenuity of the systems of investment which it offers to policy-holders. We have satisfied ourselves that, on the whole, this office offers greater advantages to professional men and others with limited incomes than probably any other insurance office in the world. Certainly hospital secretaries, especially the youngest of them, would be well advised to make themselves acquainted with the various endowments and debenture policies of the New York Mutual Life Insurance Company, and in this way they could readily provide an annuity for themselves on the best terms at a cost well within their compass.

